# Supplementary material for: Deletion of mFICD AMPylase alters cytokine secretion and affects visual short-term learning in vivo
Source: J Biol Chem. 2021 Aug 19;297(3):100991. doi: 10.1016/j.jbc.2021.100991 (PMC8441161; doi:10.1016/j.jbc.2021.100991)
Supplement: Table S2 [file mmc3.pdf]

Deletion of mFICD AMPylase alters cytokine secretion and affects visual short-term learning *in vivo*

Nicholas McCaul[1,2], Corey M. Porter[3], Anouk Becker[1], Chih-Hang Antony Tang[5], Charlotte Wijne[1], Bhaskar Chatterjee[3], Djenet Bousbaine[1,6], Angelina Bilate[1], Chih-Chi Andrew Hu[5], Hidde Ploegh[1,*] and Matthias C. Truttmann[3,4,*]

[1]Program in Cellular and Molecular Medicine, Boston Children's Hospital, Boston, MA, 02115, USA

[2]Harvard Medical School, Boston, MA, 02115, USA

[3]Department of Molecular & Integrative Physiology, University of Michigan, Ann Arbor, MI, 48109, USA

[4]Geriatrics Center, University of Michigan, Ann Arbor, MI, 48109, USA

[5]Center for Translational Research in Hematologic Malignancies, Houston Methodist Cancer Center, Houston Methodist Research Institute, Houston, TX 77030, USA

[6] Microbiology Graduate Program, Massachusetts Institute of Technology, Cambridge, MA, 02139, USA

[*]To whom correspondence should be addressed to: Dr. Matthias C Truttmann, BSRB, 109 Zina Pitcher Place, Ann Arbor 48109, MI. Phone: +1-734-615-9897; E-mail: mtruttma@med.umich.edu; ORCID ID: 0000-0002-0536-7923. Dr. Hidde L Ploegh, Boston Children's Hospital, 1 Blackfan Circle, Boston 02115, MA. Tel.: +1-617-919-1613; E-mail: hidde.ploegh@childrens.harvard.edu

SUPPLEMENTAL INFORMATION

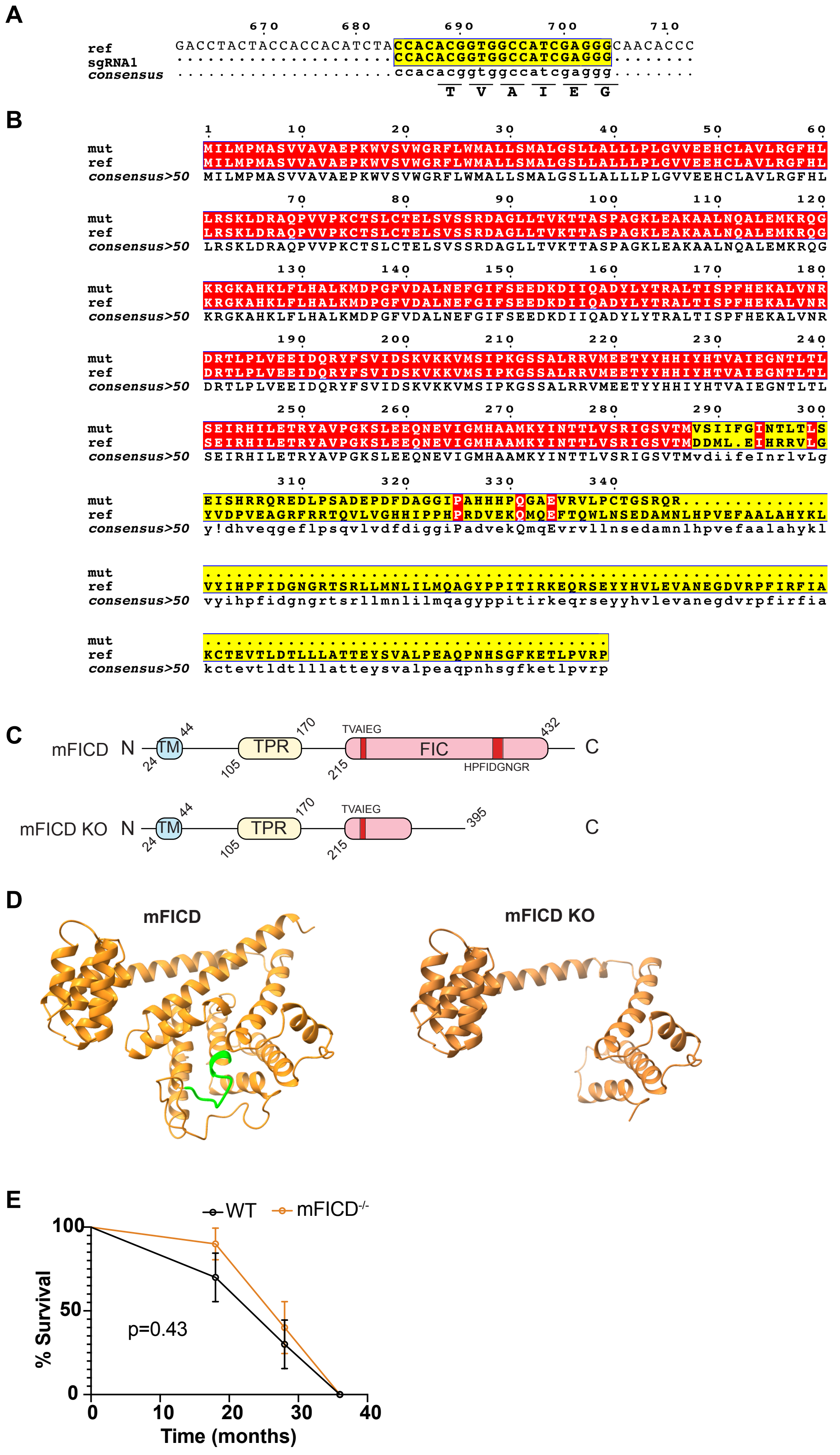

Figure S1: generation of mFICD-/- mice using CRISPR/Cas9. (A) schematic of experimental strategy. sgRNA was chosen based on its proximity to the regulatory TVAIEG motif. (B) In silico translation of mFICD-/- allele. (C) Schematic of wild-type and mFICD KO-encoded mFICD. TM: transmembrane domain; TPR: tetratricopeptide repeat domain; FIC: filamentation induced by cyclic AMP (catalytic) domain. (D) Survival of mice after 6, 16 and 28 months. Statistical significance (P=0.99) was calculated using Log rank (Mantel Cox) test.

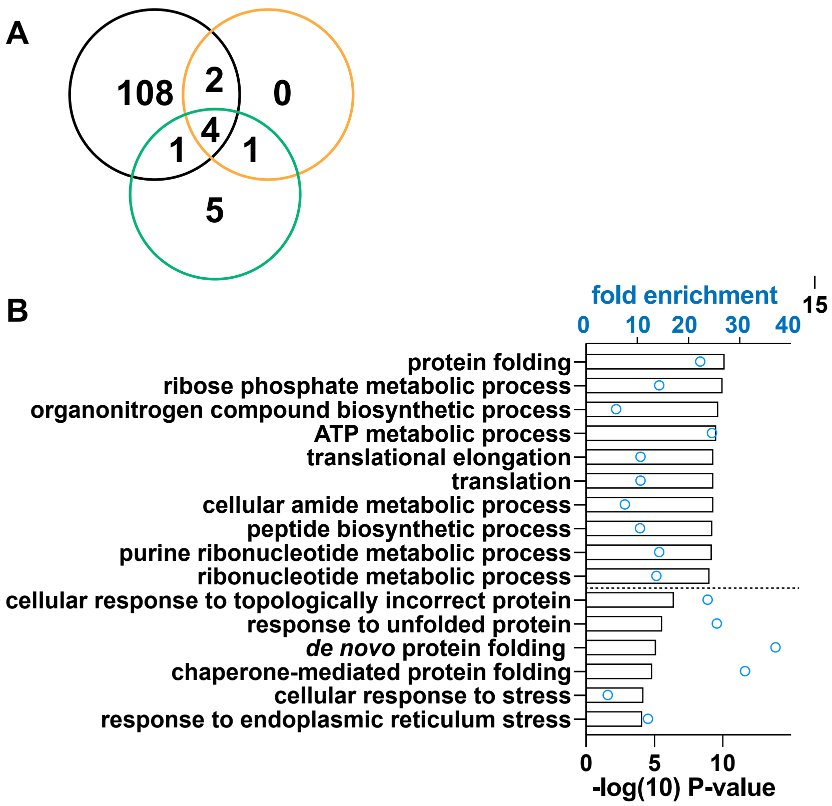

Figure S2: Gene ontology (GO) analysis of AMPylome. (A) Venn diagram showing the number of proteins AMPylated in wild-type (black circle), mFICD knockout (orange circle) MEFs and a cell-free control (turquoise circle). (B) GO enrichment analysis was performed comparing AMPylated proteins to reference proteome.

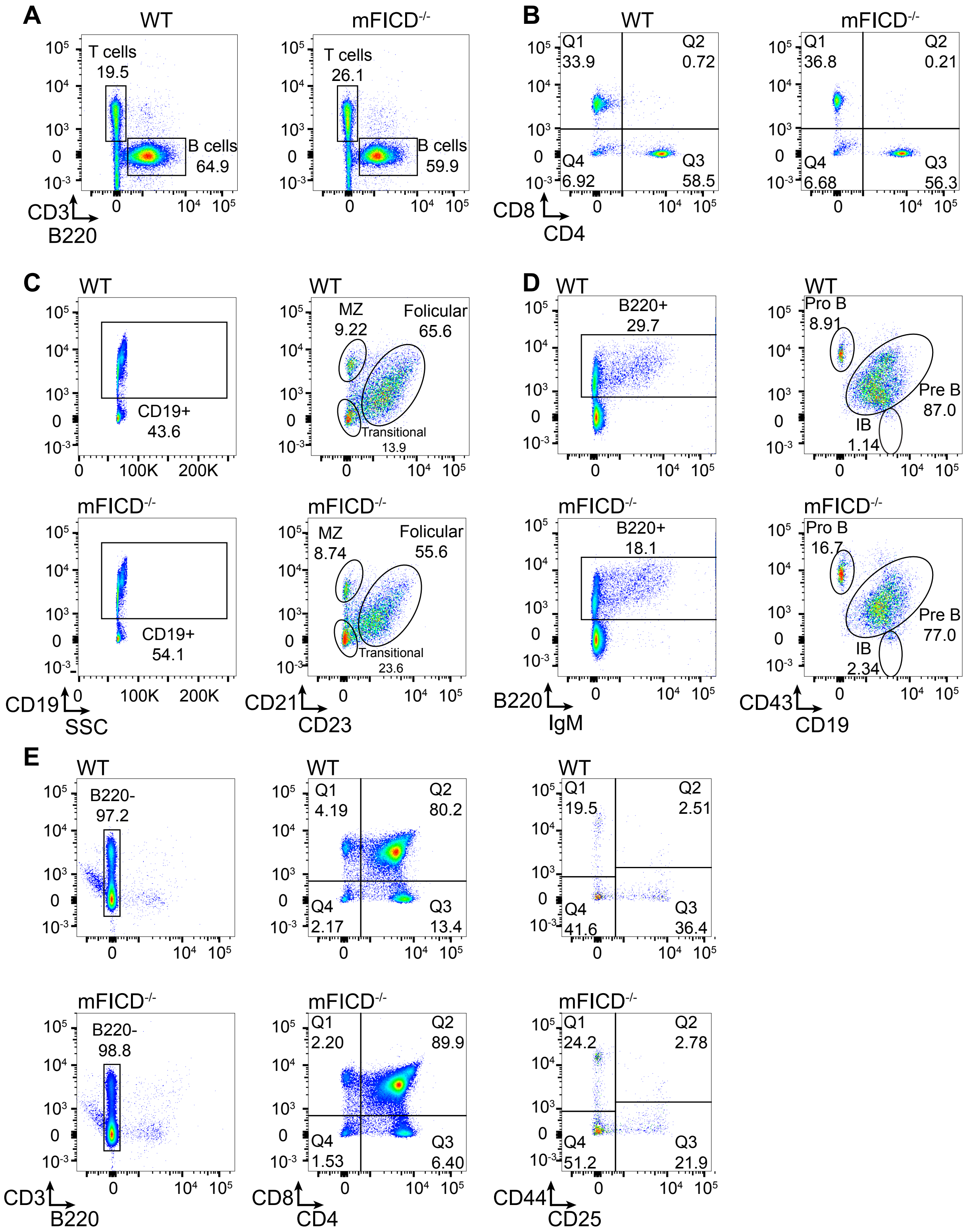

Figure S3: B and T cell development is normal in mFICD-/- mice. Splenocytes from age-matched WT and mFICD-/- mice were stained with antibodies against immune-cell markers and analyzed by flow cytometry to determine B and T cell populations (A), T cell subsets (B) and B cell subsets (C). (D) Flow cytometry was performed as in (A) on cells isolated from the bone marrow of WT and mFICD-/- mice to follow B cell development. (E) Flow cytometry was performed as in (A) on cells isolated from thymus of WT and mFICD-/- mice to follow T cell populations. (F) CD4/CD8 populations from (E) were further examined to follow T cell development. FACS plots are representative of multiple mice in each group.

Figure S4

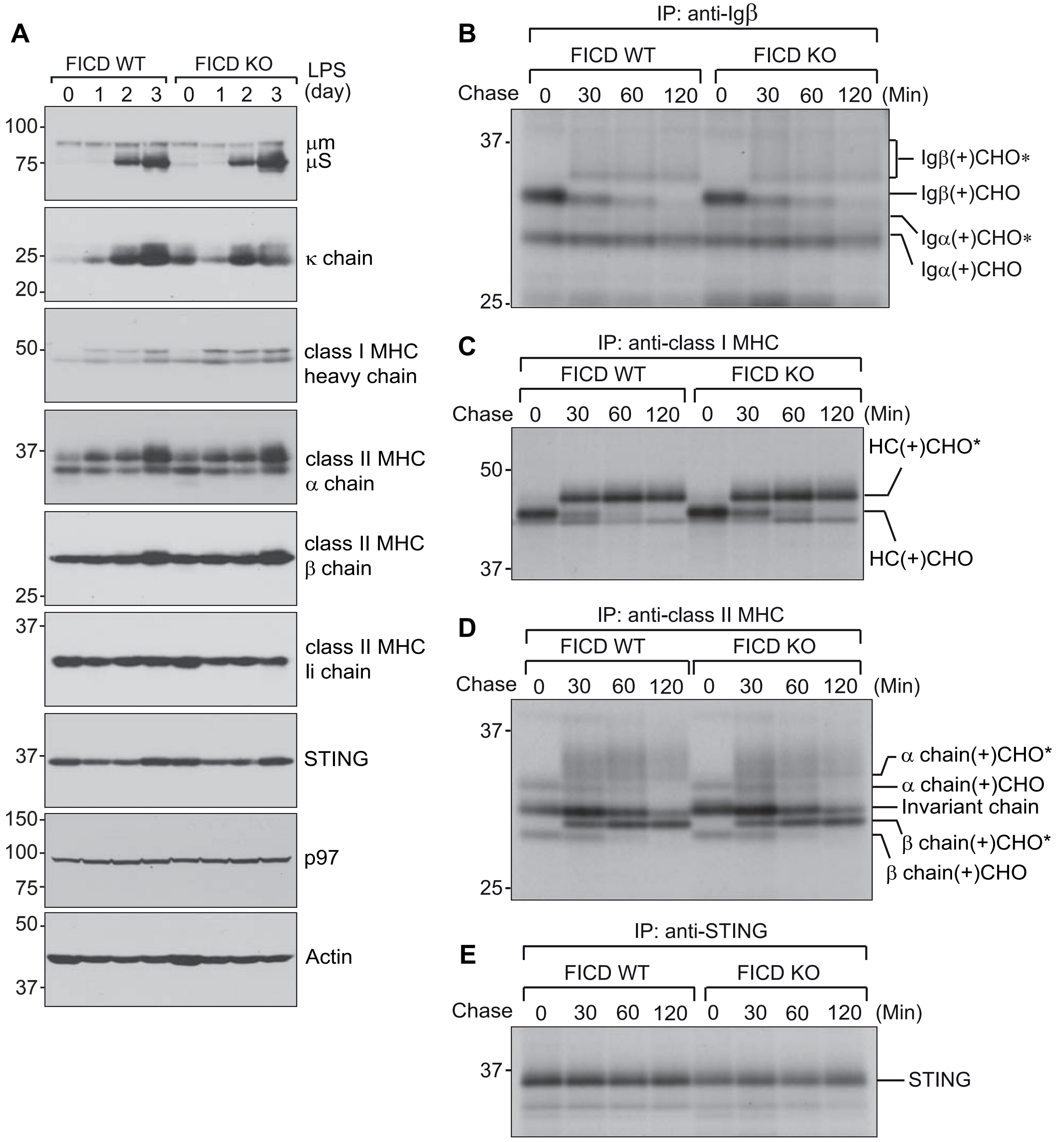

Figure S4 – Immune receptor levels and glycoprotein trafficking are normal in mFICD-/- plasmablasts. (A) Splenocytes were isolated from WT and mFICD-/- mice and incubated with lipopolysaccharide (LPS) for the indicated times. Post-nuclear supernatants were analyzed by SDS-PAGE, transferred to nitrocellulose membranes and analyzed by immunoblot for the indicated targets. (B-E) 3 day LPS-activated plasmablasts were pulsed and chased as in Figure 3D. Detergent lysates were immunoprecipitated with antibodies against Igβ (B), Class I MHC (C), Class II MHC (D), and STING (E). Immunoprecipitates were analyzed by SDS-PAGE and autoradiography.

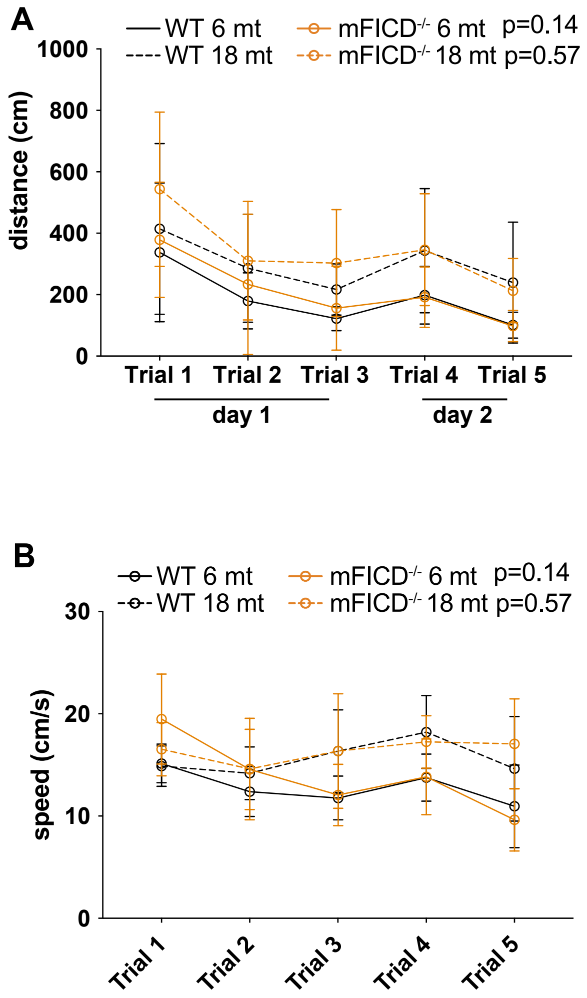

Figure S5. mFICD-/- mice show no signs of cognitive deficits. Morris water maze tests to assess visual and spatial learning and memory. (A) time to platform of 6 and 18 months old mice. (B) Average swimming speed of 6 and 18 months old animals. Statistical significance (P values) were calculated using two-way ANOVA for repeated measures with Geisser-Greenhouse correction (C). Error bars: SD.
